# Successful use of an antithrombin for heparin resistance with andexanet alfa

**DOI:** 10.1186/s40981-023-00619-7

**Published:** 2023-05-17

**Authors:** Jun Honda, Yuya Itakura, Shiori Tanaka, Satoki Inoue

**Affiliations:** Department of Anesthesiology, Fukushima Medical University Hospital, 1 Hikarigaoka, Fukushima, 960-1295 Japan

**Keywords:** Andexanet alfa, Heparin resistance, Antithrombin, Activated coagulation time, Edoxaban

To the Editor,

Andexanet alfa neutralizes the anticoagulant effects of direct factor Xa inhibitors [1]. We herein report a case in which heparin resistance occurred as a result of andexanet alfa, but thrombus formation during cardiopulmonary bypass (CPB) was prevented by antithrombin (AT) administration and monitoring both activated coagulation time (ACT) and thromboelastography (TEG).

An 81-year-old woman (155 cm, 53 kg) with a medical history of atrial fibrillation was scheduled for emergency aortic arch replacement. The patient had been taking edoxaban 30 mg/day. Although the recommended preoperative withdrawal period for edoxaban is 3 days before surgery with a risk of bleeding, there is no recommended preoperative plasma level [2].

To antagonize the therapeutic effects of edoxaban, andexanet alfa 400 mg was intravenously administered over 13 min before entering the operating room. Continuous infusion of andexanet alfa at 4 mg/min was started immediately after induction of anesthesia, which lasted for two hours in a total dose of 480 mg (Fig. 1). The ACT after induction of anesthesia was 126 s, and TEG® 6 s (Haemonetics Corporation, Boston, MA, USA) results were CK-R 5.6 min, CKH-R 5.8 min, CRT-MA 43.9 mm, and CFF-MA 9.7 mm. After induction of anesthesia, 2 g of tranexamic acid was administered. Unfractionated heparin (UFH) 30000U was administered to use CPB, but the ACT was 253 s, falling short of its target ACT. Thus, another 30000U of UFH was administered, but the ACT fell to 240 s. After completion of continuous andexanet alfa, 3000 IU of AT (Antithrombin III, Takeda Pharmaceutical Company, Tokyo, Japan) was administered after which the patient’s TEG® 6 s results were CK-R 64.2 min, CKH-R 12.2 min, CRT-MA 37.2 mm, and CFF-MA 2.2 mm. In addition, the ACT was increased to 514 s, so CPB was initiated, during which the ACT was over 999 s. After withdrawal of CPB, a total of 200 mg of protamine was administered, and the ACT returned to 185 s (Fig. 1).

**Fig. 1:**
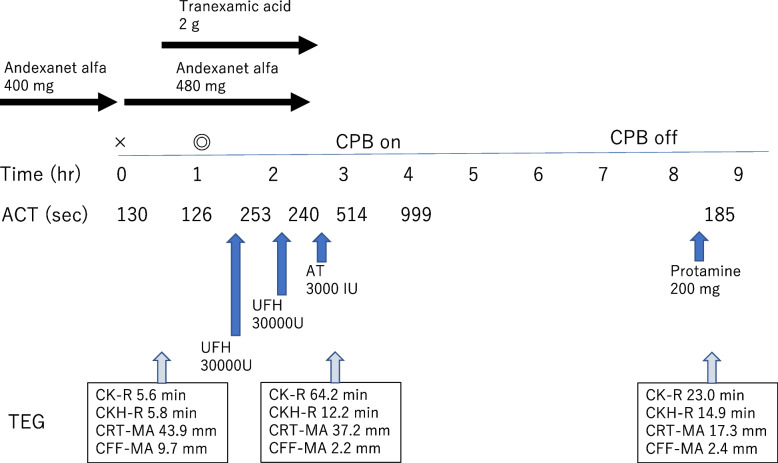
Infusion of andexanet alfa, heparin, antithrombin and coagulation parameters during anesthesia. Activated coagulation time (ACT) was 253 s following infusion of unfractionated heparin 30000 U, which was still 240 s after additional heparin 30000 U. ACT was 514 s after administration of antithrombin 3000 IU, with prolonged coagulation time measured by thromboelastography. CPB, cardiopulmonary bypass; ACT, activated clotting time; AT, antithrombin; UFH, unfractionated heparin. × : Start of anesthesia. ◎: Start of operation

Heparin resistance has been reported to occur with the use of andexanet alfa [3]. The reason for this is thought to be that andexanet alfa binds to heparin-activated AT, thereby suppressing heparin’s activity [4]. Apostel et al. reported that thrombus formation occurred in a patient with inadequate prolongation of ACT due to heparin resistance caused by andexanet alfa, but prolongation of ACT was achieved by administration of AT [5].

When performing surgery using heparin in patients who have received andexanet alfa, it is necessary to administer AT and confirm its anticoagulant effect by monitoring ACT and TEG, because of the possibility of heparin resistance.

## Data Availability

Not applicable.

## Abbreviations

CPB: Cardiopulmonary bypass
AT: Antithrombin
ACT: Activated coagulation time
TEG: Thromboelastography
UFH: Unfractionated heparin
